# Spontaneous perforation of primary gastric malignant lymphoma: a case report and review of the literature

**DOI:** 10.1186/s12957-015-0458-0

**Published:** 2015-02-08

**Authors:** Yu Ohkura, Seigi Lee, Daisuke Kaji, Yasunori Ota, Shusuke Haruta, Yasuaki Takeji, Hisashi Shinohara, Masaki Ueno, Harushi Udagawa

**Affiliations:** Department of Gastroenterological Surgery, Toranomon Hospital, 2-2-2 Toranomon, Minato-ku, Tokyo 105-8470 Japan; Department of Hematology, Toranomon Hospital, 2-2-2 Toranomon, Minato-ku, Tokyo 105-8470 Japan; Department of Pathology, Toranomon Hospital, 2-2-2 Toranomon, Minato-ku, Tokyo 105-8470 Japan

**Keywords:** Spontaneous perforation, Gastric malignant lymphoma, Distal gastrectomy, Diffuse large B cell lymphoma, Emergency, Necrotic matter

## Abstract

**Background and aims:**

Spontaneous gastric perforation in the absence of chemotherapy is extremely rare. The authors encountered a case of spontaneous perforation of primary gastric lymphoma.

**Case presentation:**

A 58-year-old man visited the authors’ hospital with acute severe epigastralgia. A large amount of free gas and a fluid collection around the stomach were noted on an abdominal computed tomography scan. The results of imaging studies indicated a perforated gastric ulcer, and a distal gastrectomy was performed. There was a large perforation about 50 mm in diameter in the anterior wall of the middle part of the stomach body. Microscopically, the full thickness of the gastric wall was diffusely infiltrated by a population of large atypical lymphoid cells. The lymphoid nature of these cells was indicated by the strongly positive immunohistochemical staining for CD20 and CD10. This confirmed the diagnosis of a germinal center B-cell-like type of diffuse large B cell lymphoma. Rituximab plus cyclophosphamide, doxorubicin, vincristine, and prednisone were administered after the operation.

**Results and conclusion:**

Gastrectomy should be considered if a giant ulcer with necrotic matter on the ulcer floor is seen on upper gastrointestinal endoscopy because of the possibility of gastric perforation. If upper gastrointestinal endoscopy shows a finding similar to the abovementioned one during chemotherapy, dose reduction of chemotherapy or gastrectomy should be considered.

## Background

Gastrointestinal non-Hodgkin lymphoma is the most common form of extranodal lymphoma. The vast majority of gastric lymphomas are extranodal marginal zone B cell lymphomas of mucosa-associated lymphoid tissue (MALT lymphoma) and diffuse large B cell lymphoma (DLBCL), which were previously considered as low-grade and high-grade gastric lymphomas, respectively [1-3]. Primary gastric lymphoma is rare, accounting for only 1%–5% of all gastric tumors [4,5].

Perforation of the gastric malignant lymphoma during chemotherapy is a well-known event. However, the incidence is not high. Furthermore, spontaneous gastric perforation in the absence of chemotherapy is extremely rare. In recent years, the standard therapy for aggressive gastric lymphoma has shifted from surgery to chemotherapy and medical therapy. Primary surgical resection is no longer the standard of care. However, it is difficult to make a preoperative diagnosis of spontaneous perforation of primary gastric lymphoma. The authors encountered a case of spontaneous perforation of primary gastric lymphoma, and it was considered in terms of pathological findings.

## Case presentation

On 6 November 2008, a 53-year-old man presented to a nearby hospital with the chief complaints of epigastric pain and black stool. Upper gastrointestinal endoscopy revealed a large deep ulceration in the gastric antrum. Enhanced abdominal computed tomography (CT) scan showed multiple enlarged para-aortic lymph nodes. The other lymph nodes were not enlarged. Positron emission tomography/CT showed abnormal accumulations of fluorodeoxyglucose in the gastric angle, para-aortic lymph nodes, pelvic lymph nodes, and prostate. They suspected malignant lymphoma or prostate cancer. The prostate biopsy showed prostate cancer, and a definitive diagnosis of gastric malignant lymphoma could not be made despite gastric biopsies that were performed every 6 months. There were no abnormal findings in the bone marrow biopsy. Because of the high-serum level of prostate-specific antigen (833 ng/ml) and prostate capsular invasion on magnetic resonance imaging (MRI), they made the diagnosis of metastasis of prostate cancer to the para-aortic and pelvic lymph nodes (cT3aN1M1). Treatment was initiated for the prostate cancer rather than the gastric malignant lymphoma. They started medical therapy for prostate cancer (bicalutamide and leuprorelin acetate). After medical therapy, enhanced abdominal CT showed that the para-aortic and pelvic lymph nodes had become progressively smaller and the level of prostate specific antigen had decreased. On the other hand, upper gastroscopy showed that the gastric tumor had enlarged gradually. Once control of prostate cancer was achieved, they had planned to start medical treatment for the gastric tumor. The presence of *Helicobacter pylori* infection was demonstrated by the positive results of histologic examination, rapid urease test, and serology. They performed *H. pylori* eradication therapy.

**Figure 1:**
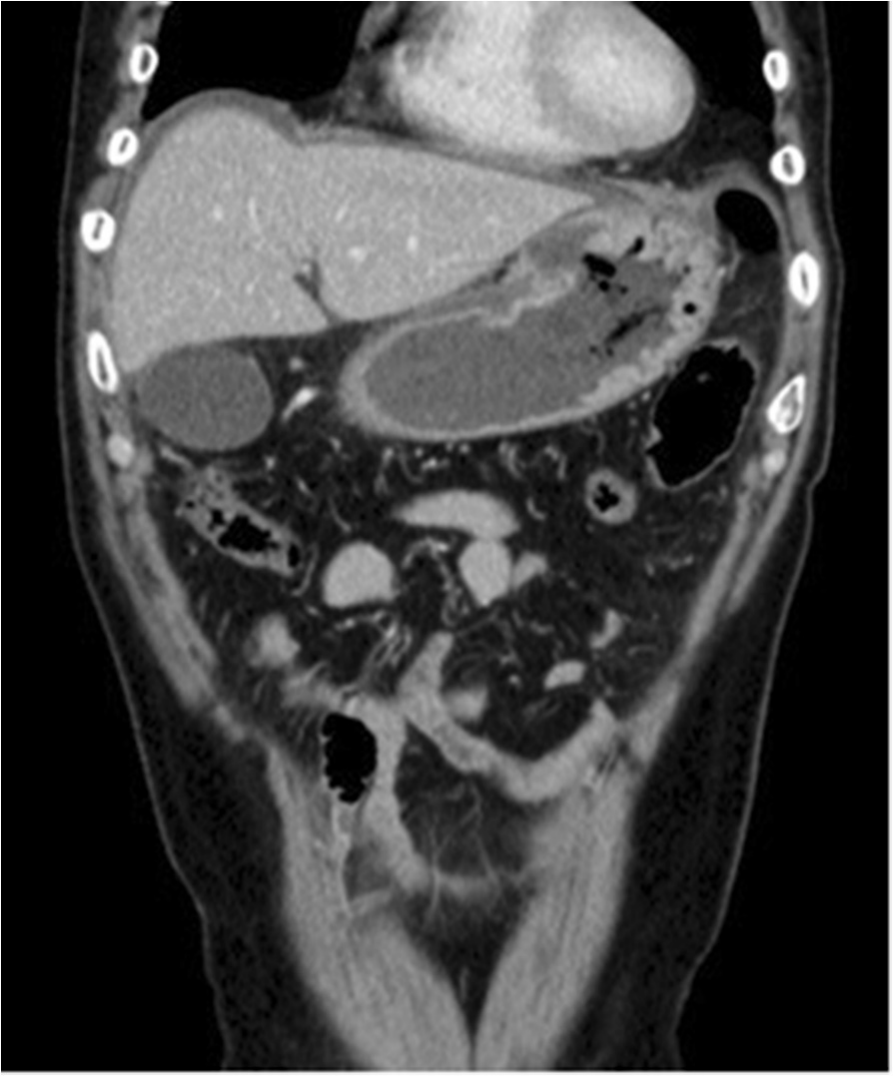
**Abdominal CT scan.** The site of gastric perforation at the anterior wall of the body of the stomach was identified. There were free gas and fluid collections around the stomach, liver, and spleen.

**Figure 2:**
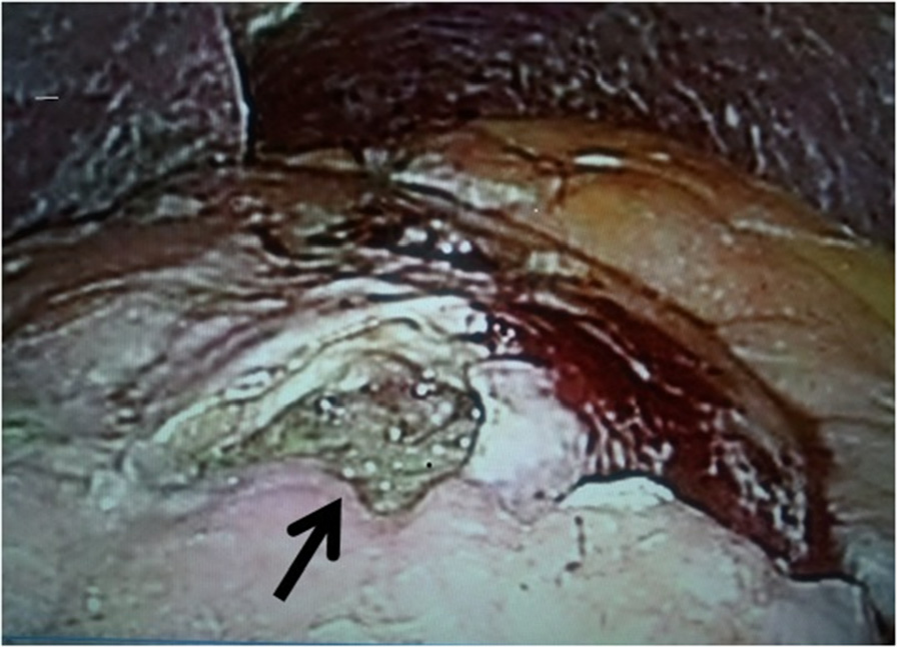
**Laparoscopic visualization of the abdominal cavity.** There was a large perforation about 50 mm in size in the anterior wall of the middle part of the stomach body (arrow) with purulent ascites and a large amount of food residue.

**Figure 3:**
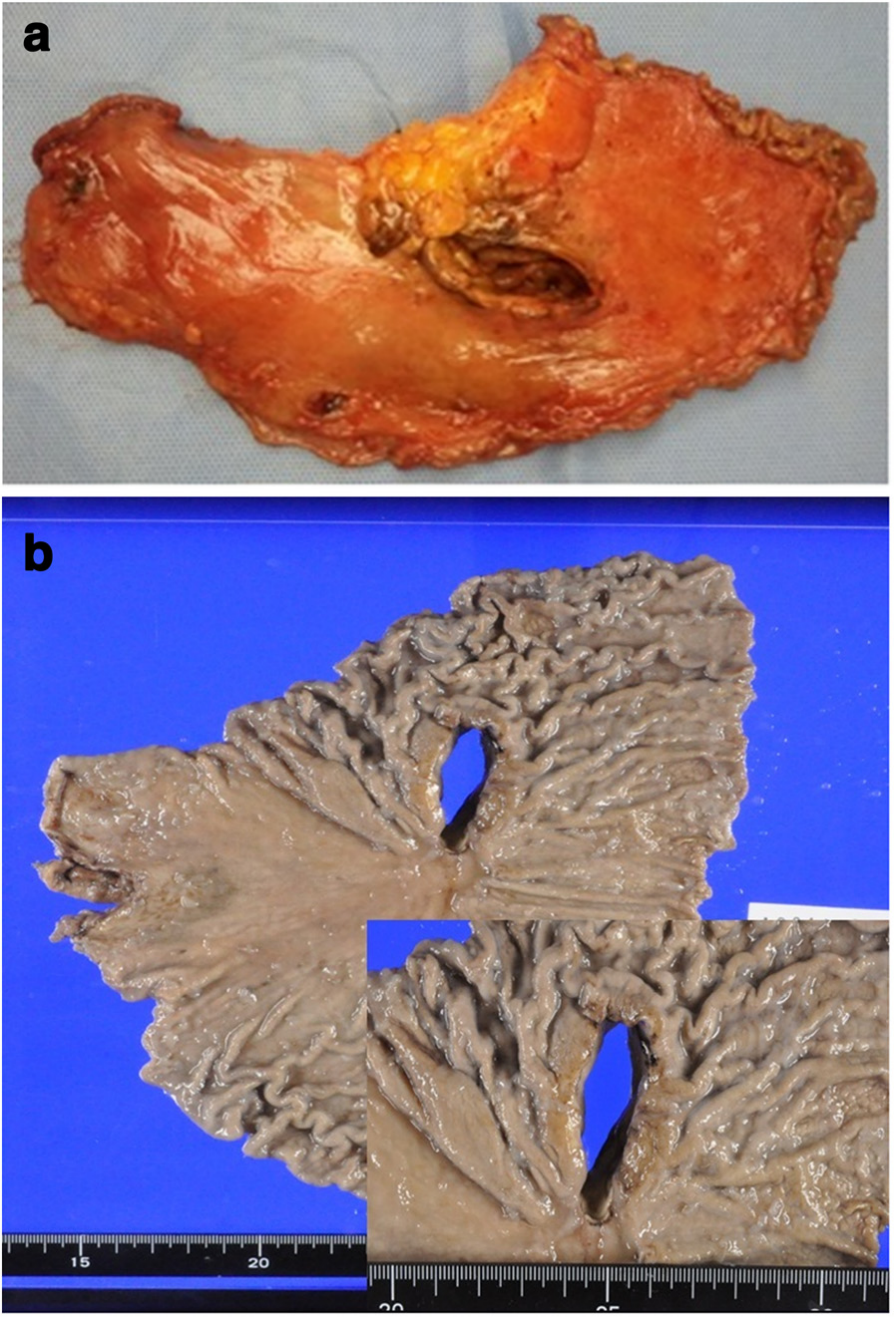
**Resected specimen and macroscopic appearance of the tumor. (a)** Resected specimen. **(b)** Macroscopic appearance of the tumor. The gastric ulcer was 4.2 × 1.5 cm in size while the perforation was 4.0 × 1.3 cm in size, and they were located on the anterior wall of the gastric antrum.

**Figure 4:**
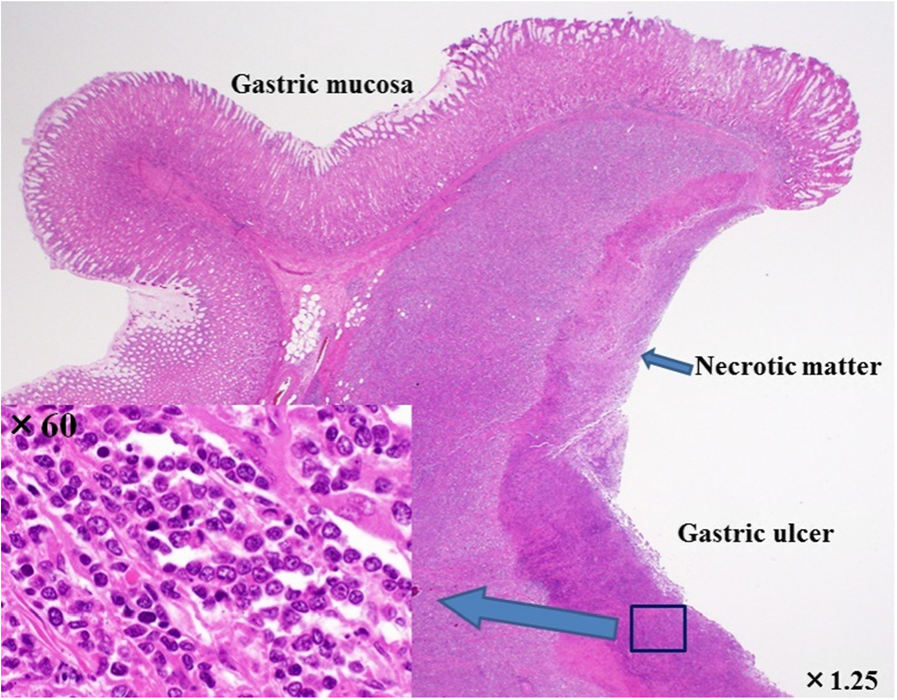
**Histological specimen.** The full thickness of the gastric wall was diffusely infiltrated by a population of large, atypical lymphoid cells. Tumor cells and necrotic matter were seen around the perforation and ulcer floor (hematoxylin & eosin staining; ×1.25, ×60).

**Figure 5:**
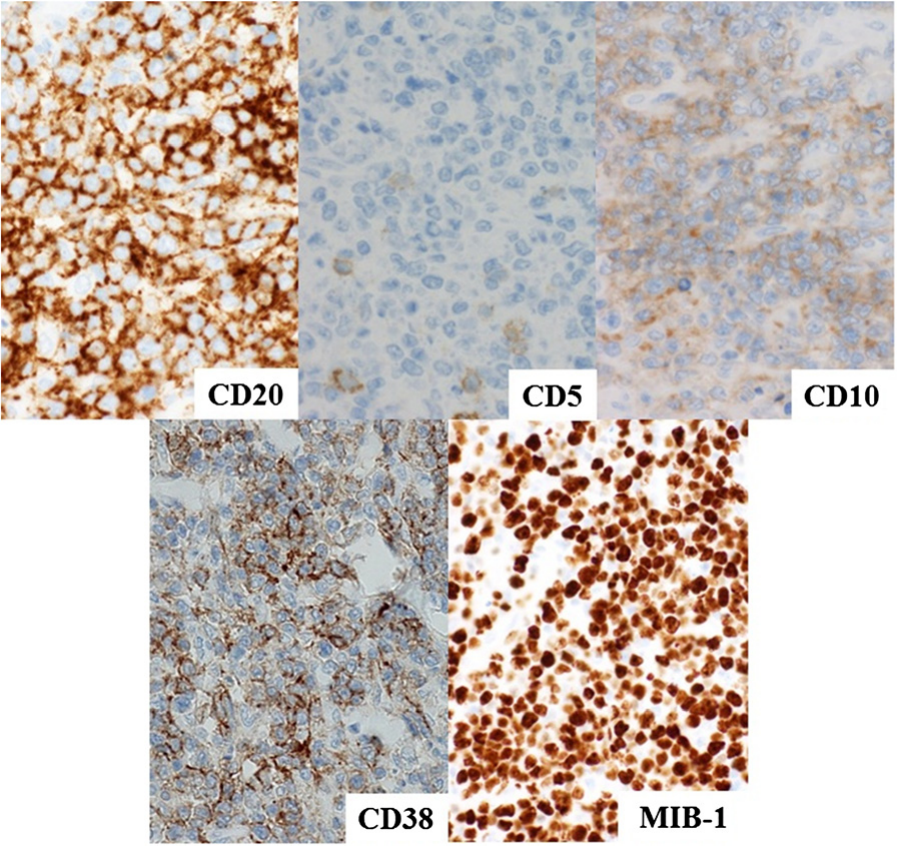
**Immunohistochemical staining.** The lymphoid nature of these cells was indicated by the strong positive immunohistochemical staining for CD20, CD38, and CD10 and negative staining for CD5. The MIB-1 labeling index was about 80% (×60).

In November 2013, the patient visited the authors’ hospital with acute abdominal pain. He had severe epigastralgia that had started after breakfast. He was 58 years old, 166-cm tall and weighed 62 kg, with a body temperature of 35.9°C, a pulse rate of 81 beats/min, and a blood pressure of 127/82 mmHg. On physical examination, abdominal distension and severe tenderness were present. Blood tests showed normal results for the white blood cell count (4,900/μl), C-reactive protein (0.1 mg/dl), lactate dehydrogenase (200 IU/l), and interleukin-2 receptor (264 U/ml). Abdominal CT scan showed a large amount of intraperitoneal free gas and a fluid collection around the stomach, liver, and spleen. Also, the site of gastric perforation at the anterior wall of the body of the stomach was identified (Figure 1). A diagnosis of perforation of gastric ulcer and pan-peritonitis was made, but details of the gastric lymphoma were unknown because it was his first visit to the authors’ hospital. The results of imaging studies indicated a perforated gastric ulcer, and an emergency operation was performed. At first, a laparoscopic omental implantation repair was planned. On laparoscopic visualization of the abdominal cavity, purulent ascites and food residue were observed. There was a large perforation about 50 mm in diameter in the anterior wall of the middle part of the stomach body (Figure 2). Because a large amount of food residue had escaped through the large perforation, removal of the food residue around and within the stomach was attempted but it was unsuccessful. Therefore, an emergency laparotomy was performed. A distal subtotal gastrectomy with Roux-en-Y anastomosis and lymph node dissection was performed. Macroscopically, the gastric ulcer was 4.2 × 1.5 cm in size, and the perforation was 4.0 × 1.3 cm in size and located on the anterior wall of the gastric antrum (Figure 3a, b). It was ulcerative and infiltrative and was the excavated type according to Sano’s classification [2]. Microscopically, a population of large atypical lymphoid cells diffusely infiltrated the full thickness of the gastric wall. Tumor cells were mainly centroblasts. Tumor cells diffusely infiltrated the muscle and subserosal layers (Figure 4). Tumor cells and necrotic matter were detected around the perforation and ulcer floor. The lymphoid nature of these cells was confirmed by the strong positive immunohistochemical staining for CD20, CD38, and CD10; the slightly positive staining for Bcl-6; and partial positivity for MUM-1 (Figure 5). On the other hand, the results for CD3, CD5, Bcl-2, and EBER ISH were negative. The MIB-1 labeling index was about 80%. These findings confirmed the diagnosis of germinal center B-cell-like type of DLBCL. There was no evidence of a low-grade MALT lymphoma. The number of metastatic lymph nodes was 3/31. Based on the Lugano International Classification, the stage was II1E (perforation and pan-peritonitis) since lymph node metastasis was present around the left gastric artery and greater curvature of the stomach and distant metastasis was absent.

He recovered from the operation uneventfully and was discharged from hospital 16 days postoperatively. He was administered rituximab plus cyclophosphamide, doxorubicin, vincristine, and prednisone (R-CHOP) therapy at the previous hospital.

## Conclusions

Malignant lymphoma of the gastrointestinal tract can be classified as nodal or extranodal. Nodal lymphomas originate in the lymphatic tissue adjacent to the gastrointestinal tract and invade it, whereas extranodal lymphomas, which are more common, originate in the gastrointestinal tract [3]. Dawson et al. [4] described five major features as the criteria for primary malignant lymphoma of the intestinal tract: 1) no palpable superficial lymphadenopathy, 2) no enlarged mediastinal lymph nodes evident on chest radiographs, 3) normal total and differential white blood cell counts, 4) prominent bowel lesions at laparotomy, and 5) no tumors in the liver or spleen. The patient had a large ulcer in the stomach, and infiltration of other organs was not found. The present case met all of the abovementioned criteria, so this case was considered to be primary gastric lymphoma. Primary gastric lymphoma is rare, accounting for only 1%–5% of all gastric tumors [5].

In recent years, the standard treatment for aggressive gastric lymphoma has shifted from surgery to chemotherapy. The treatment varies with the histology of the malignant lymphoma. First-line chemotherapy for DLBCL of the stomach is CHOP with or without rituximab. On the other hand, antibiotic treatment to eradicate *H. pylori* is the first-line therapy for MALT lymphoma. Bayerdolffer et al. [6] reported that about 70% of patients showed complete regression and about 12% had partial regression of lymphoma but 18% had no change after eradication of *H. pylori* infection. Radiation treatment for *H. pylori*-negative gastric MALT lymphoma has a high-success rate of 90% or better after 5 years. Also, there were some reports strongly supporting the hypothesis that some gastric *de novo H. pylori*-positive DLBCL might remain *H. pylori*-dependent and are therefore responsive to *H. pylori* eradication therapy [7,8]. In the present case, the presence of *H. pylori* infection was indicated by the positive results of the histologic examination, rapid urease test, and serology. Despite *H. pylori* eradication therapy in the present case, there was no size reduction of the gastric malignant lymphoma.

Primary gastric lymphoma often presents with nonspecific symptoms, and the diagnosis is often delayed. Nonspecific abdominal pain (50%) and dyspepsia (30%) are the most common presentations. B symptoms (fever, night sweat, and weight loss) are uncommon in contrast to nodal lymphomas; thus, the diagnosis might be delayed [9].

It is widely known that perforation occasionally occurs in patients receiving chemotherapy. Yoshino et al. [10] and Maisey et al*.* [11] reported that perforation of gastric lymphoma in patients receiving chemotherapy occurs in about 0.9% to 1.1% of cases. On the other hand, spontaneous perforation of malignant gastric lymphoma is rare compared with perforation of gastric lymphoma in patients receiving chemotherapy. Table 1 shows 15 cases of spontaneous perforation of primary gastric lymphoma that required gastrectomy in Japan between 1985 and 2013 [3,12-24]. Of these 15 patients, nine were men and six were women with an age range from 22 to 91 years and a mean age of 61.4 years. The mean tumor size was 91.3 mm (15–200 mm), and the mean diameter of the perforation was 14.5 mm (3–40 mm). Patients with spontaneous perforation of primary gastric lymphoma requiring gastrectomy had larger tumors and perforations. The present patient had the largest perforation out of those in past reports.

**Table 1:** **Literature reviewed**

**Case**	**Author**	**Year**	**Age**	**Sex**	**Location**	**Tumor size (mm)**	**Diameter of perforation (mm)**	**Sano’s classification**	**Operation**	**Pathology**	**Lugano**	**Adjuvant chemotherapy**
1	Kanzaki et al. [12]	1985	42	Male	U	32	5	Excavated	DG	Burkitt	II1E	+
2	Ando et al. [13]	1992	22	Male	L	15	ND	Ulcerative	DG	DLBCL	IIE	+
3	Yanagi et al. [14]	1992	65	Male	L	185	25	Excavated	DG	DLBCL	II1E	+
4	Shiomi et al. [15]	1997	71	Male	M	150	20	Excavated	TG	DLBCL	II2E	+
5	Fukuda et al. [16]	1998	45	Male	M	90	7	Excavated	DG	DLBCL	II1E	+
6	Miyamoto et al. [17]	1999	46	Male	ML	30	6	Ulcerative	DG	MALT	IIE	−
7	Yabuki et al. [3]	2000	53	Male	M	100	10	Excavated	DG	DLBCL	IIE	+
8	Mori et al. [18]	2005	65	Female	U	30	ND	Ulcerative	TG	DLBCL	IV	+
9	Tanaka et al. [19]	2007	84	Female	M	90	ND	Excavated	DG	DLBCL	II2E	+
10	Matsunaga [20]	2008	73	Male	M	135	3	Ulcerative	TG	DLBCL	II1E	+
11	Saito et al. [21]	2010	67	Female	ML	85	ND	Excavated	DG	DLBCL	IIE	+
12	Ishimaru and Kitsukawa [22]	2011	54	Female	M	200	5	Excavated	TG	DLBCL	II1E	+
13	Sunagawa et al. [23]	2011	91	Female	M	120	8	Excavated	DG	DLBCL	II1E	−
14	Shimada et al. [24]	2013	85	Female	L	65	30	Ulcerative	TG	DLBCL	II1E	−
15	Present case	2013	58	Male	M	42	40	Excavated	DG	DLBCL	II1E	+

*U* upper, *M* middle, *L* lower, *ND* no description, *DG* distal gastrectomy, *TG* total gastrectomy, *DLBCL* diffuse large B cell lymphoma, *MALT* mucosa-associated lymphoid tissue lymphoma.

The cause of perforation of gastric lymphoma in cases receiving chemotherapy is different from those in cases that did not receive chemotherapy. Ono et al*.* [25] reported that the causes of perforation in patients receiving chemotherapy are weakening of the gastric tissue associated with rapid tumor necrosis, tumor lysis, and exuberant granulation due to chemotherapy. On the other hand, Shiomi et al*.* [15] reported that there are two different patterns of spontaneous perforation. First, the spontaneous perforation results from an ulcer and tumor necrosis that has reached the subserosa. Second, the perforation results from an ulcer that has thin connective tissue with the absence of tumor. In the present case, tumor cells and necrotic matter were seen microscopically around the ulcer floor and perforation site, so it was considered that spontaneous perforation resulted from the existence of an ulcer and necrosis of the tumor that had reached the subserosa.

It is difficult to make a preoperative diagnosis of spontaneous perforation of primary gastric lymphoma. In recent years, the standard treatment for aggressive gastric lymphoma has shifted from surgery to chemotherapy. However, gastrectomy should be considered if a giant ulcer and necrotic matter on the ulcer floor are present on upper gastrointestinal endoscopy because of the possibility of gastric perforation. If upper gastrointestinal endoscopy shows a finding similar to the abovementioned one during chemotherapy, dose reduction of chemotherapy or gastrectomy should be considered.

In conclusion, a case of spontaneous perforation of primary gastric malignant lymphoma was reported and the literature was reviewed. Further research and examination of similar cases are required to confirm this relationship (the indication of surgery and pathological findings), and further assessment of the clinical significance of spontaneous perforation of primary gastric lymphoma is needed.

## Consent

Written informed consent was obtained from the patient for publication of this case report and any accompanying images. A copy of the written consent is available for review by the Editor-in-Chief of this journal.

## References

[1] Khadraoui H, Feigin KN, Fox JJ, Onq L, Shike M, Yahalom J (2013). Successful management of gastric perforation in Stage IV diffuse large B-cell lymphoma with chemoradiation therapy, percutaneous endoscopy gastrostomy for gastric drainage, and percutaneous endoscopy jejunostomy for nutrition. Clin Lymphoma Myeloma Leuk.

[2] Sano R (1987). Classification of malignant gastric lymphoma.

[3] Yabuki K, Tamasaki Y, Satoh K, Maekawa T, Matsumoto M (2000). Primary gastric lymphoma with spontaneous perforation: report of a case. Surg Today..

[4] Dawson IM, Cornes JS, Morison BC (1961). Primary malignant lymphoid tumors of the intestinal tract: report of 37 cases with a study of factors influencing prognosis. Br J Surg..

[5] Ghai S, Pattison J, Ghai S, O’Malley ME, Khalili K, Stephens M (2007). Primary gastrointestinal lymphoma: spectrum of imaging findings with pathologic correlation. Radiographics..

[6] Bayerdörffer E, Neubauer A, Rudolph B, Thiede C, Lehn N, Eidt S (1995). Regression of primary gastric lymphoma of mucosa-associated lymphoid tissue type after cure of Helicobacter pylori infection. MALT Lymphoma Study Group. Lancet.

[7] Kuo SH, Yeh KH, Wu MS, Lin CW, Hsu PN, Wang HP (2012). Helicobacter pylori eradication therapy is effective in the treatment of early-stage H pylori-positive gastric diffuse large B-cell lymphomas. Blood..

[8] Ferreri AJ, Govi S, Radere M, Mule A, Andriani A, Caracciolo D (2012). Helicobacter pylori eradication as exclusive treatment for limited-stage gastric diffuse large B-cell lymphoma: results of a multicenter phase 2 trial. Blood..

[9] Brooks JJ, Enterline HT (1983). Primary gastric lymphomas: a clinicopathologic study of 58 cases with long-term follow-up and literature review. Cancer..

[10] Yoshino S, Nakamura S, Matsumoto T, Konomi H, Hirahashi M, Yao T (2006). A case of primary gastric malignant lymphoma perforated immediately after administration of chemotherapy. Jpn Gastroenterol Dis.

[11] Maisey N, Norman A, Prior Y, Cunningham D (2004). Chemotherapy for primary gastric lymphoma does inpatient observation prevent complications?. Clin Oncol..

[12] Kanzaki M, Yokoyama T, Saito Y (1985). A case of perforated gastric malignant lymphoma. J Tokyo Womens Med Coll.

[13] Ando O, Sato T, Umeda A (1992). A rare case of gastric malignant lymphoma which was diagnosed due to perforation. J Nihon Univ Med Assoc..

[14] Yanagi S, Kouya K, Kudo Y (1992). A case of perforated malignant gastric lymphoma. Donan Med..

[15] Shiomi H, Watanabe E, Umeda T (1997). A case report of perforated gastric malignant lymphoma. Jpn J Canc Clin..

[16] Fukuda N, Tachibana A, Yamakawa T, Sakai S (1998). A case of gastric malignant lympnoma with gastric perforation. J Jpn Soc Clin Surg..

[17] Miyamoto K, Shimizu Y, Inada K, Ikeda T, Futamura N (1999). Mucosa-associated lymphoid tissue lymphoma with perforation of the stomach- A case report-. Jpn J Canc Clin.

[18] Mori H, Shirai Y, Kamiya T (1996). A case of perforated malignant gastric lymphoma. Jpn Abd Emerg Med..

[19] Tanaka T, Iwasa M, Haneda H (2007). A case of malignant lymphoma with spontaneous gastric perforation. Jpn J Gastroenterol Surg..

[20] Matsunaga M (2008). Successful treatment of primary gastric lymphoma diagnosed as tumor perforation. J Jpn Soc Clin Surg.

[21] Saito T, Nozawa S, Nagai H (2010). A case of malignant lymphoma diagnosed with spontaneous tumor perforation. J Jpn Soc Clin Surg.

[22] Ishimaru A, Kitsukawa M (2011). Report of a case of perforated giant gastric malignant lymphoma. Jpn J Canc Chemother.

[23] Sunagawa M, Isogai M, Hanada T (2012). Gastric malignant lymphoma with spontaneous perforation. Jpn J Canc Chomother.

[24] Shimada S, Gen T, Okamoto H (2013). Malignant gastric lymphoma with spontaneous perforation. BMJ Case Reports..

[25] Ono K, Matsumura S, Sakamoto K, Kobayashi S, Kamano T, Iwasaki R (1997). A case of gastric malignant lymphoma with perforation during chemotherapy. Gan To Kagaku Ryoho.

